# Editorial: Recent advances in application of synthetic biology for production of bioactive compounds, volume II

**DOI:** 10.3389/fbioe.2024.1517610

**Published:** 2024-11-14

**Authors:** Luan Luong Chu, Jae Kyung Sohng, Hanhong Bae, Dipesh Dhakal

**Affiliations:** ^1^ National Key Laboratory of Enzyme and Protein Technology, University of Science, Vietnam National University, Hanoi (VNU), Hanoi, Vietnam; ^2^ Department of Life Science and Biochemical Engineering, SunMoon University, Asan-si, Chungnam, Republic of Korea; ^3^ Department of Pharmaceutical Engineering and Biotechnology, SunMoon University, Asan-si, Chungnam, Republic of Korea; ^4^ Department of Biotechnology, Yeungnam University, Gyeongsan, Gyeongbuk, Republic of Korea; ^5^ Department of Medicinal Chemistry, University of Florida, Gainesville, FL, United States

**Keywords:** synthetic biology, bioactive compounds, metabolic engineering, crispr cas, engineered microbes

Bioactive compounds are the substances that are found in medicinal plants, bacteria, fungi and marine organisms. Both natural and unnatural bioactive compounds include secondary metabolite and their derivatives, such as isoprenoids, isoflavonoids, peptide antibiotics, and glycoside derivatives of alkaloids. These compounds play a significant role in various fields including pharmaceutical and agrochemical products, cosmetics, biofuels, and food additives. The extraction and isolation of natural products from living organisms have played an important role in the production of medicines. Along with nature bioactive compounds, synthetic biology has been developed to produce natural and non-natural compounds. This Research Topic provides recent advances, emerging challenges, and prospects of synthetic biology for bioactive compounds.

Along with model microbes (*E. coli* and *Saccharomyces cerevisiae*) that have been used for the production of natural bioactive compounds, non-conventional hosts have been developed for the biosynthesis of industrial products. Rojo et al. demonstrated the advantages of *Escherichia coli* and *S. cerevisiae* compared to plant-based production systems. The yields of isoflavonoids derivatives pterocarpans and coumestans from plant species are usually low and time-consuming industrial products are required. To overcome these limitations, engineered microbes have been used as an alternative approach to boost the production titer of pterocarpans and coumestans. Giménez et al. reviewed that filamentous fungi have been developed as a novel platform in the biotechnology field. The most advantageous filamentous fungi include the ability to grow on many different substrates and plant residues, which have a key contribution to the circular bioeconomy. According to the well-known whole-genome sequence of filamentous fungi, *Agrobacterium tumefaciens*-mediated transformation and the GoldenBraid (GB) modular cloning platform [FungalBraid (FB) (https://gbcloning.upv.es/fungal/)] have been developed to adapt to the production of bioactive compounds. The use of FB toolkit, including CRISPR/Cas system, has been easily adapted to fungi, given the interchangeability of DNA parts between GB and FB systems. Interestingly, Fathy et al. showed that microalgae including *Synechocystis* sp. PAK13 and *Chlorella variabilis* DT025 are emerging hosts for industrial production from sustainable feedstock. This is because microalgae have substantial advantages compared to traditional microbial production systems, such as high photosynthesis efficiency, metabolic versatility, rich metabolite content, CO_2_ sequestration, efficient lipid production, and more. Moreover, it is demonstrated that *Synechocystis* sp. PAK13 and *C. variabilis* could increase the accumulation of malic acid, amino acids, and indole-3-acetic acid using glycine as both a carbon and nitrogen source. Intestinally, the fatty acid content significantly increased by 1.36-fold in *Chlorella* and 2.5-fold in *Synechocystis* in the presence of glycine. Glycine is a cost – effective feedstock for microalgal cultivation, offering key benefits such as a nitrogen source, enhancing photosynthesis, reducing CO_2_ dependency, and increasing lipid and biomass production.

Expanding the structural diversity of bioactive compounds through biosynthetic methods has resulted in the formation of novel compounds. These compounds are expected to improve metabolic stability and biological activitives. Lu et al. efficiently obtained 22 new analogs with modified 5′-aminouridine moieties through mutational biosynthesis of *SsaM* and *SsaK.* Both genes are responsible for biosynthesis of the 5-aminouridine moiety of sansanmycin *in vivo*. As a result, SS-KK-2 exhibited better antibacterial activity against *E. coli* ∆tolC than the parent compound sansanmycin A. Moreover, SS-KK-3 not only showed significantly increased structural stability but also retained the sama anti-TB activity against *M. tuberculosis* H37Rv compared to sansanmycin A. On the other hand, the glycoside derivative demonstrated that it is a promising alternative to the production of natural and unnatural bioactive compounds. Heo et al. identified two glycosyltransferase-coding genes, *kid7* and *kid21,* in the kidamycin biosynthetic gene cluster (BGC) of *Streptomyces* sp. W2061 strain. While Kid7 first attached N, N-dimethylvancosamine to the C_10_ position of angucycline aglycone, Kid21 transferred an anglosamine moiety to C_8_ of the C_10_-glycosylated angucycline in the following step. These catalyzation resulted in di-C-glycosylated angucycline, which is an unusual C-glycosylated residue. Similarly, Yang et al. studied the glycosylation of rebaudioside D (Reb D). A selective glycosyltransferase (UGT94D1) and its mutants (UGT94D1-F119I/D188P) from *Sesamum indicum* catalyzed for glycosylation of Reb D, leading to the production of a mono β-1,6-glycosylated derivative, Reb M2. Notably, this mutant showed a 6.33-fold improvement in catalytic efficiency and produced Reb M2 with 92% yield (29.79 mg/mL). Therefore, these studies provide an efficient method for the future development of synthetic derivatives.

Gene expression is a critical strategy for producing bioactive compounds in microbial cell factories. Understanding the key enzymes of biosynthetic pathways, as well as their complex regulation, is necessary to improve the production of bioactive compounds. Huang et al. summarized the role of (E)-4-hydroxy-3-methylbut-2-enyl pyrophosphate (HMBPP) reductase (IspH) in the methylerythritol phosphate (MEP) pathway for the synthesis of isoprenoid compounds from engineered microorganisms. Moreover, novel catalytic activities and potential biological applications have been suggested as part of the significant role of IspH. Traditional methods, including chemical inducers and genetic modifications, have been successfully used to increase gene expression levels. However, the development of novel approaches for obtaining gene expression data is still required to control the timing and location of gene expression. Yamazaki et al. suggested the novel light-controlled gene expression and proposed a label-free light control approach using mid-infrared and terahertz light. This method allows for photocontrol of gene expression without any photosensitive molecular tags. It is expected that light-controlled gene expression exhibits the potential for groundbreaking innovations in the field of gene expression, contributing to medical and pharmaceutical development. Additionally, the modulation of enzyme spatial distribution is an essential strategy to increase enzyme activity and reduce the loss of intermediate metabolites. Liu et al. reviewed the development and employment of artificial scaffold systems in microbial cell factories. Synthetic scaffolds are based on proteins, nucleic acids, and various organelles. The approaches played a significant role in increasing the titers of bioactive compounds in *E. coli*, *Bacillus subtilis*, and *S. cerevisiae*.

In summary, recent research has shown the importance of synthetic biology in the production of bioactive compounds. Noticeably, Wang et al. insisted on the influence of synthetic biology from natural sciences to humanities and social sciences by introducing biosafety, biosecurity, and ethical issues to society. Along with the development of synthetic biology tools (CRISPR/Cas, artificial scaffold systems, omics technology, and machine learning (ML) platforms), ML-based synthetic biology-combined artificial intelligence (AI) has promising applications for improving the yield of bioactive compounds from industrial microbes as well as non-conventional hosts. Artificial and intelligent hosts are expected to combine carbon-fixing autotrophs and heterotrophs along with a high yield of bioactive compounds. It is believed that engineered microbes play a significant role in agricultural and medical biotechnology, with net zero greenhouse gas emissions.

